# Tricky ticks: the importance of Lyme carditis recognition

**DOI:** 10.1007/s12471-015-0745-y

**Published:** 2015-09-09

**Authors:** J.A. Jansweijer, R.J. van Oort

**Affiliations:** Department of Experimental Cardiology, Academic Medical Center, Amsterdam, The Netherlands

Lyme disease, or Lyme borreliosis, is caused by spirochetes of the *Borrelia burgdorferi* sensu lato species and is the most common tick-borne infectious disease in Europe and North America [1]. A characteristic early presentation of the disease is erythema migrans, an expanding erythematous skin lesion, which typically occurs days to weeks after the tick bite. Without early treatment, the *Borrelia* spirochete can disseminate leading to infection of other parts of the skin, joints, nervous system and heart.

Lyme carditis, which results from direct invasion of the heart by spirochetes, can lead to conduction disturbances and possibly myocarditis and pericarditis [2]. The most frequent clinical manifestation is atrioventricular (AV) block of varying degrees, which can fluctuate and progress rapidly from first- to third-degree block [3]. Third-degree AV block, or complete heart block, is the most severe form, in which AV electrical dissociation leads to a slow ventricular rhythm. If not managed and treated appropriately, this can trigger life-threatening ventricular tachycardia and ventricular fibrillation. Although temporary cardiac pacing may be required in up to a third of Lyme carditis patients, rushing the implantation of a permanent pacemaker is not recommended as treated patients usually show complete recovery [2]. Obviously, it is of great importance to properly diagnose Lyme carditis. This is especially relevant since there is a continuing increase in tick bites and Lyme disease in the Netherlands [4].

Hofhuis et al. performed a broad survey on physician-reported incidence of different Lyme disease associated manifestations [5], of which the results on Lyme carditis are presented in this issue of the *Netherlands Heart Journal* [6]. The authors leveraged the fact that every person in the Netherlands is registered with only one general practitioner (GP). In daily practice, a GP consultation is required for a patient to be referred to a medical specialist in the hospital and the specialist will report back to the GP. Taking this into account, Hofhuis et al. first sent a retrospective questionnaire about Lyme borreliosis diagnoses in the years 2009 and 2010, including clinical diagnoses of Lyme carditis, to all GPs in the Netherlands. The response rate to the question on Lyme carditis was 33 % among GPs, representing 46 % of the Dutch population, and yielded 39 reported cases of Lyme carditis. This accounts for 0.2 % of all GP-reported Lyme borreliosis related diagnoses [5].

Since the diagnosis of both Lyme borreliosis and Lyme carditis can be rather complex, the next step of the study consisted of a systematic review of the medical records of the reported Lyme carditis cases. Not all GPs, however, were able to cooperate with this step and ultimately only 11 medical records were available for review. Of these, 2 records were mistakenly marked as Lyme carditis by the GPs and another single record was reported twice by 2 GPs working in partnership. Finally, the diagnoses of the 8 remaining cases were evaluated by excluding other possible causes for the cardiac symptoms and by categorisation into various degrees of likelihood of Lyme carditis, based on self-designed diagnostic criteria. This way, the authors found that 6 of the reviewed medical records satisfied their criteria for a very likely Lyme carditis diagnosis, 1 satisfied their criteria for a likely Lyme carditis diagnosis and 1 was categorised as not Lyme carditis. Then, taking into account only the very likely and likely diagnoses, the crude incidence rate was adjusted, resulting in an annual Lyme carditis incidence rate of 6 (95 % CI: 4–8) per 10 million inhabitants of the Netherlands in 2009 and 2010. This gave an estimated total number of 10 Lyme carditis cases diagnosed nationwide per year in 2009 and 2010.

Several factors in this study may lead to an underestimation or overestimation of the real Lyme carditis incidence rate, amongst which the low response rates of GPs to the questionnaires and medical record review, and the sometimes difficult diagnosis of Lyme borreliosis. Furthermore, cardiac conduction disturbances are relatively common in the population, which may lead to an overestimation of the incidence rate, although the exclusion of other causes of cardiac conduction disturbances and the required combination with other diagnostic criteria should at least largely prevent this overestimation.

In conclusion, Lyme carditis is a rare manifestation of Lyme borreliosis, albeit potentially grave and even life-threatening. Also, if unrecognised as Lyme carditis, total AV block may lead to the unnecessary implantation of a pacemaker. Considering the increase in Lyme borreliosis diagnoses in past years, the study presented by Hofhuis et al. is a timely reminder to all physicians, and GPs in particular, that Lyme carditis should be included in the differential diagnosis when assessing cardiac symptoms. This is especially the case in the presence of atrioventricular conduction disturbances, an absence of apparent risk factors and a history of recent exposure to ticks.

## Funding

R.J.v.O. is supported by a VENI grant (916.11.150) from the Netherlands Organization for Scientific Research (NWO) and a Dr. Dekker Senior Postdoc grant (2012 T094) from the Dutch Heart Foundation (NHS).

## Conflicts of interest

None declared.
